# Editorial Work and the Peer Review Economy of STS Journals

**DOI:** 10.1177/01622439211068798

**Published:** 2021-12-30

**Authors:** Wolfgang Kaltenbrunner, Kean Birch, Maria Amuchastegui

**Affiliations:** 1 Leiden University, Leiden, the Netherlands; 2 York University, Toronto, Ontario, Canada

**Keywords:** peer review, gift economy, scholarly publishing, editorial work, political economy

## Abstract

In this paper, we analyze the role of science and technology studies (STS) journal editors in organizing and maintaining the peer review economy. We specifically conceptualize peer review as a gift economy running on perpetually renewed experiences of mutual indebtedness among members of an intellectual community. While the peer review system is conventionally presented as self-regulating, we draw attention to its vulnerabilities and to the essential curating function of editors. Aside from inherent complexities, there are various shifts in the broader political–economic and sociotechnical organization of scholarly publishing that have recently made it more difficult for editors to organize robust cycles of gift exchange. This includes the increasing importance of journal metrics and associated changes in authorship practices; the growth and differentiation of the STS journal landscape; and changes in publishing funding models and the structure of the publishing market through which interactions among authors, editors, and reviewers are reconfigured. To maintain a functioning peer review economy in the face of numerous pressures, editors must balance contradictory imperatives: the need to triage intellectual production and rely on established cycles of gift exchange for efficiency, and the need to expand cycles of gift exchange to ensure the sustainability and diversity of the peer review economy.

## Introduction

Journal peer review is a mechanism for constituting and legitimating the publishability of scholarly manuscripts. It is also increasingly framed as a problem of supply and demand of review labor in an academic environment that incentivizes authorship over other kinds of scholarly work, especially activities that are not made visible through any formal accounting (Fox, Albert, and Vines 2017; Gropp et al. 2017). The analysis presented in this paper will contribute to a better understanding of how transformations in the organization of journal publishing and, in particular, increased competition for publishing space affect the various epistemic control functions peer review is meant to fulfill. As an analytical inroad to this problem, we will describe and unpack the daily work carried out by journal editors in our own field of science and technology studies (STS). Editors have a special role in the peer review process (Crane 1967; Hackett and Chubin 1990; Tennant and Ross-Hellauer 2020; Vermeir 2020). Among other things, they are usually responsible for screening manuscripts upon submission and for ensuring a steady supply of committed reviewers who can offer informed and constructive feedback in a timely manner. We will argue that these tasks involve careful organization and management of an imperfect scholarly gift economy (Mauss 2016), which runs on perpetually renewed experiences of mutual indebtedness.

Attending to the complexities of editorial work in the organization of peer review is particularly topical because the broader conditions under which peer review is conducted are in transition. A pertinent long-term trend is, first, the growing importance of metrics like the journal impact factor (JIF) and associated authorship practices aimed at maximizing publication output in high-impact journals (Fochler 2016). Citation-based metrics entail a quantitative hierarchy of journals that potentially undermines previously more organic experiences of mutual obligation in an intellectual community. Simultaneously, STS is growing as a field, while also differentiating into a large array of publication outlets. But when scholarly dissemination becomes more spread out across journals, the peer review economy is likely altered, too (de Solla Price 1962). Equally relevant to our analysis are changes in the funding models of scholarly publishing and the growing market dominance of a handful of commercial publishers (Larrivière, Haustein, and Mongeon 2015). Editors are dependent on commercial partnerships because services and infrastructure provided by publishers significantly facilitate the operation of a journal once it surpasses a certain volume of submissions. Yet reliance on such infrastructure and the move to individually paid article processing charges (APCs) can be expected to reconfigure the social relationships on which the peer review economy depends (Posada and Chen 2018; Vann 2017; Horbach 2019).

The empirical basis of our analysis is a set of 76 semi-structured interviews with informants from the STS publishing world, speaking from different actor positions: as authors, reviewers, editors in various functions, and publishers. Informants are associated with a range of STS journals, including general and specialist outlets. The scope of the material allows us to compare editorial strategies in dealing with pressures on the peer review system, thereby providing input for ongoing discussions about the timeliness and problems of editorship in STS journal publishing.

## The Peer Review System as a Gift Economy

Peer review has most commonly been studied in terms of its effectiveness in evaluating or falsifying scientific claims. Such literature is often produced by scientific practitioners with a personal interest in addressing shortcomings of peer review; for example, failure to detect misconduct or bias (Abramowitz, Gomes, and Abramovitz 1975; Peters and Ceci 1982; Weller 2002). Rather than focusing on aberrations from a posited epistemological norm, STS scholars have tended to analyze peer review as constitutive of and legitimating particular forms of interaction between authors, editors, and reviewers (Hackett and Chubin 1990; Hirschauer 2010, 2015; Pontille and Torny 2014; Horbach and Halffman 2020; Horbach 2019; Eve et al. 2021; Siler and Strang 2017).

Despite these efforts, the political–economic organization of peer review remains understudied, both in regard to the labor it draws on and in terms of how scholarly publishing as a business mediates its epistemic workings. A few noteworthy exceptions deserve mention. The classic study by Zuckerman and Merton (1971) interrogated the distribution of review responsibilities within particular intellectual communities, analyzing which reviewers are assigned to review certain papers and the questions this raises for fairness and objectivity. In an instructive historical case study, Fyfe et al. (2020) charted the quantitative growth of peer review labor and the concomitant need to expand the community of reviewers for the *Philosophical Transactions of the Royal Society* from 1865 onward. And Horbach (2019, 190-210) studied the implications of a partial delegation of certain review tasks—such as screening manuscripts in terms of robustness and scientific interest—to commercial actors.

In this paper, we build on this literature by focusing on the work done by editors in organizing and curating the peer review economy. Peer review is a quintessential form of “invisible labor” that is often presupposed, but whose actual organization is not usually analyzed. A common notion among academics is that peer review is the collective responsibility of members of an intellectual community, fueled by the exchange of favors among individuals who rely on each other’s unpaid work to pursue shared goals. In short, an economy based on gift exchange as theorized in the influential writing of Marcel Mauss (2016).

The idea that scientific work is based on gift-giving has a tradition in the sociology of science (Hagstrom 1965, 1982; Kelty 2001; Vermeir 2013; see also Bergquist and Ljungberg 2001). Building on Mauss, Hagstrom (1965, 1982) argued, for example, that scientific papers themselves are a form of gift. Hagstrom suggested that scientists are driven by a desire for recognition as members of a scientific community and that they publish primarily to adhere to the communal expectation that one should share one’s work freely. Built into this interpretation of the gift is obviously a strong functionalist emphasis on norms as the glue of scientific communities (Merton 1942; Polanyi 1969; Lievrouw 1989). While scientists are depicted as wanting to be recognized for their contribution, a shared ethos of communalism and disinterestedness here is seen to override individual self-interest or careerism. In Hagstrom’s account, peer review labor is conceptualized more in passing as a further service to the community that scientists provide in order to be recognized as community members.

In our view, it is questionable whether the circulation of publications at large could ever be usefully understood as a gift economy, given that the convertibility of publications into reputation, employment, and funding opportunities connects publishing rather closely to a logic of accumulation (Latour and Woolgar 1986; Fochler 2016; Hessels et al. 2019). We argue that it makes more sense to think of the communal provisioning of review labor in a more restricted sense as a gift economy.

Ever since the widespread adoption of journal peer review in the mid 20th century, it has been a common expectation among members of intellectual communities that previously published authors repay their debt for the labor a journal has invested in their manuscripts by acting as reviewers for others (Treviño 2008). As classic contributions to the sociology of science have pointed out (Ziman 2000; Zuckerman and Merton 1971), members are expected to take responsibility for reviewing in areas of research they themselves specialize in, and senior scholars are seen as having a particularly strong obligation toward their community because of the proportionally higher volume of reviews (i.e., gifts) they have received. Authors are also expected to show commitment toward the journal they choose to submit a manuscript to in the first place. Most online submission systems require authors to confirm that their manuscript is not currently under review elsewhere. Among other things, journals thereby aim to prevent the labor invested in editing and reviewing a manuscript from going to waste when authors decide to take their work to another outlet. Finally, editors are seen as obliged to authors, reviewers, and readers of a journal in equal measure. They must ensure that submissions are properly considered and given a “fair hearing,” while making good use of reviewers’ voluntary labor to select and improve manuscripts. The position of the editor is itself often conceived as a marker of intellectual esteem, which simultaneously commits editors to further service to their community (Fyfe and Gielas 2020).

A crucial implicit assumption in popular accounts of the review gift economy is that it is based on a stable community, meaning, first, that the population of researchers submitting manuscripts to a journal neatly coincides with its readership and, second, that individual researchers constantly cycle through the different roles—author, reviewer, and sometimes editorial team member. These two preconditions ensure that obligations are experienced from different perspectives and thus are more likely to be upheld (Ziman 2000; Polanyi 1969).

In our analysis, we draw on these insights as starting points for analyzing peer review as a gift economy. Feelings of mutual obligation clearly are key constituent elements of a field, perhaps equally important as shared conceptual frameworks and research methods. Yet, if taken too literally, the notion of peer review as a gift economy tends to simplify this economy to the point where it appears as a self-regulating system that is fueled by shared norms and inherent dynamics of reciprocity. Both functionalists like Hagstrom and certain techno-utopian accounts of scholarly publishing (cf. Vermeir 2020) have a tendency to reduce the legitimate role of editors to what is essentially an administrative job: it should avoid any subjective judgment and limit itself to managing flows of gift exchange that would occur anyway, given researchers their contributions. In our own empirical analysis, we show that the peer review gift economy is significantly less stable and self-organizing, requiring a constant curating effort by editors. Moreover, community relations, we argue, are increasingly *the product* of this curatorial work rather than its stable substrate.

## The Role of Editors in Organizing and Maintaining a Peer Review Gift Economy

Characteristically, from the perspective of authors, many functions fulfilled by editors are ill-understood and even interpreted as arbitrary. And while inevitably involving subjective judgment, editorial work becomes analytically more tangible if we acknowledge that all the crucial intellectual decisions it entails have a political–economic dimension as well.

The editorial process starts with the initial screening of manuscripts. Screening serves to assess whether a manuscript is sufficiently developed and an epistemic fit for the outlook of a journal. Simultaneously, it is crucial to keep in check the sheer number of manuscripts the journal has to actively handle at any given time. This part of editorial work remains invisible to authors unless they receive a desk-rejection.

As a next step in the process, editorial work entails selecting and inviting suitable researchers to review a manuscript. It is in fact common for authors to complain about being mismatched with reviewers (Silbiger and Stuber 2019); for example, on the grounds of insufficient expertise or lack of sympathy for a particular intellectual approach. Such match-making indeed implies significant leeway for editors, but it must again be seen as configured by political–economic limitations. More specifically, it is constrained by a crucial curatorial activity in the background, namely the need for editors to actively cultivate a pool of committed reviewers. A common experience for editors is dealing with reviewers who are unreliable or unresponsive. Reciprocity in gift exchange does not happen automatically; instead, it must be evoked and organized by editors. One of the more visible manifestations of the effort required are annual open letters in which editors list otherwise anonymous reviewers by name and thank them for their service (e.g., Hackett, Ribes, and Vann 2019). While the transactional character of gift exchange normally remains implicit (Mauss 2016), this very act serves to remind researchers of their responsibility to the intellectual community. Reviewers should moreover be epistemically diverse enough to cover a broad range of papers, especially in the case of general journals. Nothing guarantees, however, that an otherwise unregulated or unorganized gift exchange would produce such an intellectual diversity.

Finally, editors must report review outcomes to authors and decide whether further investment of journal resources is warranted. This entails a process of commensurating review reports (Espeland and Stevens 1998; Lamont 2012); that is, a meta-evaluation to assess the usefulness of evaluative opinions and so render them comparable. Editors must also help authors make sense of the reports without damaging any of the involved relationships—the relationship with authors in case of a rejection, but also the one with reviewers in case their recommendation is overridden.

The job profile of editors is thus more complex than implied by functionalist and popular views of the academic journal publishing system (cf. Csiszar 2018; Fyfe et al. 2020). It requires not just channeling, but actively organizing and managing feelings of reciprocal obligation on the part of researchers who are routinely overworked and incentivized to focus on a specific range of accountable activities (such as writing, acquiring grants, teaching). Aside from such inherent complexity, there are various challenges that arise from the entanglement of review work with the broader conditions of scholarly publishing.

The peer review gift economy is in fact embedded in various other economies, for which it helps provide the material infrastructure. First, the economy around scholarly reputation, employment, and research evaluation, where publications are a form of capital (Fochler 2016; Hammarfelt 2017). Second, the epistemic discourse of STS, which amounts to an economy of producing and trading intellectual contributions—be it novel empirical knowledge, interesting critique, societal engagement, original theoretical work that can be reused by others, and so on. Finally, the political economy of scholarly publishing as a business. We theorize that the obligation dynamics on which peer review gift-giving is based can become resources in these embedding economies, and vice versa (cf. Åkerstöm 2017). Relationships built through gift exchange can, for example, influence substantive evaluative decisions in peer review. As such, they are sometimes pursued strategically, as when researchers accept review invitations from a journal to put themselves on favorable terms with its editor(s). But personal rapport can also be seen more innocuously as a lubricant that helps members of a community to better understand each other’s contributions and intellectual context. The historical absence of formal monetary transaction in the shape of fees or rewards for authors can be seen as a precondition for the very emergence of gift exchange as an economic modality for organizing peer review in the first place.

We also suggest that the relationships between gift-giving in peer review and its encompassing economies were relatively stable throughout the second half of the 20th century, thus allowing researchers to get used to the dynamics emerging from their intertwinement. However, these relationships are being reconfigured due to various ongoing, momentous, shifts.

First, historically, the growing importance of indicators like the JIF combined with increased competition for academic employment encourages researchers in the publishing ecosystem to more explicitly think like investors (Fochler 2016; Birch and Muniesa 2020). Although even commercial publishers nowadays caution against uncritical reliance on metrics (Taylor and Francis n.d.), especially early career academics across fields tend to react to career uncertainties by trying to publish at an increasing rate and ideally in journals whose reputation is formally acknowledged across academic communities through metrics like the JIF (Sigl 2016; Nästesjö 2021). Editors in turn are encouraged to act as stewards of their journal’s reputation, whose worth can now be more formally gauged in terms of citation rates and article downloads. These trends have likely played a role in rendering the commitments that constitute intellectual communities more conditional on anticipated gains than used to be the case before. And since more time spent on writing papers means less time for reviewing, they also appear to promote a systemic disproportion between volume of submissions and supply of review labor.

Moreover, the constitutive feelings of obligation that underpin the review economy are likely affected by the growth and differentiation of the intellectual production of STS as a field (de Solla Price 1962). In small fields, social relations and experiences of mutual indebtedness can be expected to be tight by default (Whitley 2000; Becher and Trowler 2001). However, such bonds will tend to weaken as fields grow and the number of journals increases. This is partly due to the lack of opportunities for individuals to interact in more personal ways, either through physically meeting at conferences or through informal exchanges in the context of submission processes. Increase of formal communication channels like journals will also go along with a tendency toward epistemic differentiation into self-contained specialties, since representatives of competing approaches are no longer forced to address each other in the same limited set of publication venues.

Another pertinent development is the changing commercial and research funding models that underpin scholarly journal publishing in STS. For a long time, there was an unquestioned commercial structure based on subscription bundles paid by libraries (Fitzpatrick 2011), which allowed researchers to largely ignore the economics of publishing. The growing market dominance of a small number of publishers and the spread of new commercial funding models have recently begun to disrupt this arrangement. On one hand, partnering with large corporate publishers allows editors to make use of their digital infrastructure and other services to facilitate the production side of scholarly publishing. Publishers in turn try to reduce the marginal costs of producing articles by scaling up infrastructures across journals according to a platform logic (Vann 2017; Posada and Chen 2018; Horbach 2019), with unclear effects for social interactions reliant on intellectual community structures. The move to the research funding model based on open access (OA), which is based on APCs paid by authors, also affects feelings of personal indebtedness by transforming publishing into a more commodity-like transaction. The payment of APCs raises questions as to whether authors should still feel obliged to review if they already paid for an article and also how community relations are reconfigured if OA is reserved for authors with sufficient funds (Squazzoni, Giangiacomo, and Károly 2013; Zaharie and Osoian 2016).

In the following section, we briefly describe the methods we relied on to collect and analyze interview data on the strategies STS journal editors pursue to organize and maintain a peer review gift economy in the face of such diverse pressures. The subsequent empirical analysis outlines these strategies in significant detail, with our narrative being structured according to the main steps of the editorial process: screening manuscripts, assigning reviewers, drawing together review reports, and making decisions about acceptance.

## Methods and Data Collection

This study draws on semi-structured interviews with 74 STS scholars and two representatives of commercial publishers. Based in different geographical regions (Table 1), all academic informants have experience as both authors and reviewers, and 21 of them are editors or associate/managing editors responsible for handling submissions.

**Table 1.** Distribution of Academic Informants According to Geography

Geographical Region	Academic Informants
Asia	5
Australia	5
Europe	35
North America	27
Latin America	2
*n*	74

Our academic informants are associated with 10 STS journals, which represent a sample of both well-established and more recently founded journals, as well as outlets with a specific thematic or geographical focus (Table 2). For each journal, we interviewed researchers who have published in the respective outlet, current editors and associate/managing editors, as well as researchers on the editorial/advisory boards. The latter review regularly and tend to have a good idea of the editorial process of a journal, although they usually do not have an executive editorial function. Note that individual informants may have multiple roles in different journals. Since we operated under the premise of full confidentiality, we will use the undifferentiated label “editorial team #” when referencing quotes from informants with any editorial responsibilities.

**Table 2.** Informants in Editorial Positions According to Journals

	Editorial Team Members
*Social Studies of Science*	13
*Science, Technology, & Human Values*	9
*Science as Culture*	11
*Social Epistemology*	3
*Tapuya: Latin American Science, Technology and Society*	7
*Catalyst: Feminism, Theory, Technoscience*	8
*East Asian Science, Technology and Society: An International Journal*	10
*Engaging Science, Technology and Society*	6
*Science & Technology Studies*	5
*Valuation Studies*	1

We conducted the interviews between November 2019 and June 2020. Focused on practices of writing, editing, and reviewing scholarly literature, they ranged between 45 and 120 minutes and were transcribed in full. We analyzed the transcripts using the Nvivo qualitative data analysis software package (Release 1.0). More specifically, we coded the material according to iteratively refined categories that focus on diverse aspects of editorial work and the peer review process, for example, “becoming an editor,” “recruiting reviewers,” and “role of metrics.”

## The Curatorial Work of Editors

### Screening Manuscripts

Journals do not exist in isolation but position themselves vis-à-vis co-existing and partly competing publishing outlets. Within the field, two of the oldest STS journals are widely considered particularly important: *Social Studies of Science* and *Science, Technology, & Human Values*. Yet several of our respondents referred to a perception of there not being enough journals for STS scholarship, and the existing journals not providing enough room for more specific debates. A number of relatively recent journals, in fact, position themselves in explicit contradistinction or complementarity to the established journals; for example, *Valuation Studies*, *Catalyst*, or *Engaging Science, Technology and Society*. Another important element in shaping the profile of a journal in recent years is the inclusion in the Web of Science and assignment of a JIF. High JIFs have a significant effect on submissions, with the most visible journals witnessing a doubling or even tripling of submissions in recent years. At least some of these submissions are likely at the direct expense of non-indexed journals. The emergence of such contingent journal profiles is relevant to our analysis because it heavily configures the responsibilities of their editors.

A major challenge for editors with highly ranked journals, which are often disciplinary “core” journals, is to manage the corresponding increase in editorial and review labor through thorough initial screening and desk rejections of manuscripts. As one informant (editorial team #67) explained:

[T]he number of submissions to [the journal] increases steadily, not fast but steadily every year. (…) That [current figure is] about 50% more than we were receiving when I started as [editor] [several] years ago (…) so we have been aiming for a higher desk-reject rate but that is not exactly related to the movement of the field. (…) I have been wanting to have in play at any one time, a certain number of manuscripts, because otherwise it gets overwhelming for me and the editorial team. But it also becomes challenging to find reviewers.

To understand the high ratio of desk rejections in the most coveted journals—often around 50 percent (editorial team #67)—one must appreciate that reviews are rarely one-off activities. Rather, a first round of reviews often induces a longer process of re-reviewing, with unclear outcome. Sending out a manuscript for review thus constitutes a significant commitment of resources and time.

Screening simultaneously serves an affective function in managing various relationships. On one hand, editorial screening must demonstrably honor the effort of the author even in the case of desk rejections. These should be justified, however briefly and without unnecessary harshness, because authors are always potential reviewers. Many editors therefore make sure to carefully reread desk rejection letters before sending them out to submitting authors. Screening is also a crucial practice for cultivating and maintaining a pool of committed reviewers. Reviewers may feel disrespected if they are assigned clearly underdeveloped or otherwise unsuitable manuscripts. The result may be careless or overly critical review reports, as well as a likelihood that the reviewer will think twice before accepting another review assignment in the future. One informant (editorial team #56) put it in particularly drastic terms:

So, what we try to do is protect all of our reviewer pool from reading lousy papers. (…) [A colleague of mine] (…) stopped reviewing for one journal because they felt that they were always being asked to take out the trash. They would send them just lousy papers and it would be, like, their responsibility to give them a justification for turning it down. And so while I am a little concerned about making that decision so far upstream, the alternative is that the reviewer pool will dwindle.

Another informant (editorial team #65) noted that, as a result, “papers that are either less well developed or more [out on the] margins of STS [may] not [be] getting the chance to get feedback from reviewers, essentially.” This points to a paradox in a field like STS, which sits at the margins of many disciplines. As submissions increase, for whatever reason, editors seek to organize and manage a fragile peer review gift economy at the risk of a retrenchment of disciplinary boundaries. It is, then, important to consider in more detail the criteria editors apply in screening manuscripts.

A first criterion could be described as an attempt to discern the intentions and sincerity of the submitting authors. Some submissions are preceded by a long-standing history of previous exchanges and personal acquaintance between authors and editors. However, an increasingly common situation is that submitting authors have little apparent prior knowledge of STS, sometimes picking a journal specifically because of its high JIF. Yet another possibility is that authors submit to a journal primarily to get reviews and test the waters but are not willing to revise the paper if it doesn’t immediately meet the approval of the referees. Many authors appear to operate not with a specific journal in mind, but with a hierarchical list of journals that they “slide down” until they get accepted, thereby minimizing waste in their production of articles. One of our informants suggested that the growth of the journal landscape combined with career uncertainties especially for younger scholars facilitates such a productivity-oriented approach:

[T]here are more places you can submit to, so it almost kind of lowers the stakes of getting a rejection. And if I get one from *Big Data & Society*, maybe I can submit to *Social Media + Society* (…) that’s not a great way of going about writing quality work necessarily, but it’s an option for people who want to quickly publish papers. (Author and reviewer #40)

Digitalization of publishing further limits personal interaction, for example, by making it optional for authors to include a letter justifying the rationale for submission. At the same time, authors sometimes actively evoke personal obligation in the submission process. Some editors reported that some authors bypass the online submission platform by sending a personal email with a manuscript in the attachment. This is partly done to check whether an article is suitable, but partly also in the hope that personal interaction will increase the chances of surviving editorial screening (editorial team #67).

Editorial screening is of course also informed by more specific epistemic criteria, which are fraught with questions of indebtedness and reciprocity in their own right, albeit in ways that are more akin to trading than gift-giving. Especially editors of general journals expressed a sense of obligation to acknowledge and represent the intellectual traditions of STS, while ensuring enough room for submissions that innovate in terms of analytical frameworks, critical outlook, or research topics. This “essential tension” (Kuhn 1977; Hackett 2005) between old and new points to the historicity of STS. In the early days of the field, most published research was by definition pioneering. But over time, STS has built up a body of literature that authors can be seen to trade with, in the sense of drawing on existing insights and frameworks and ideally making some kind of appropriate return. Editors of general journals police the fairness and suitability of this trading and thereby actively define the boundaries of the field. One informant (editorial team #56) interpreted their role as follows:

Trying to stitch together the fabric of our field, and not let each year be something completely different. The next new habit. So I talked about this being double gated. So one side of the gate is like the new stock, trying to find a way to let in the new stock. Even if maybe the reviews are not that strong. And the other is to try and avoid becoming captives to one or another perspective, for lack of a better word.

The transactional character becomes particularly clear in cases where editors feel that authors make opportunistic use of STS references.

Another boundary that I end up drawing is (…) when I get papers from adjacent disciplines that are drawing on the STS literature, but (…) in a way that really speaks to their own home discipline and doesn’t speak to STS…I’ll see a lot of papers where it’s, like, people discovering actor-network theory or, kind of, the power of materiality (…) but when it comes to our journal it doesn’t really advance the conversation within STS because people are going to look at that and be like, “hell yes, materiality matters.” (Editorial team #65)

The funneling of manuscripts toward highly visible journals also directly affects screening practices in less established outlets. Fewer submissions and less visibility across academic communities generally reduce the need for screening manuscripts upfront, sometimes limiting it to a very basic check of adherence to formal guidelines. Also, there is more room for “working with the author.” The editor of a more recently established journal emphasized their willingness to provide a sounding board for researchers who do not have access to informed feedback from colleagues at their home institutions (editorial team #70). At first sight an altruistic gesture, such extra effort also constitutes a special form of expectation toward the submitting author, namely that their submission can thereby be turned into a particularly robust contribution that will help consolidate the status of the journal.

Differentiation of the journal landscape can also promote a form of selective obligation on the part of authors. Some journals intentionally abstain from seeking indexation in the Web of Science, either to avoid the various concessions required as part of the indexation process, or as part of a political choice not to participate in what one informant called the “impact factor machine” (editorial team #64). Such positioning amounts to a powerful self-selection criterion for authors, thus creating and consolidating communities that define themselves by opposition to intellectual mainstreams. An example is the journal *Catalyst*, which is meant to provide an outlet that is theoretically and politically sympathetic to explicitly feminist STS scholarship. The decision to submit a manuscript is often preceded by informal communication through which authors exchange their views “about what journals are supportively run and what journals are unsupportively run” (editorial team #64). As a result, the journal does not attract many submissions by authors who are not already familiar with relevant debates. A more focused journal profile thus makes it easier for editors to organize and manage the peer review gift economy.

The subjective judgment inherent in screening manuscripts raises the question of how responsibility for this important editorial task is distributed. Several informants indicated that they aim to avoid concentrating too much authority in a single individual (editorial team #75, #56) and that screening should ideally be carried out by two or more members of the editorial team (and, if possible, from different career stages). Feasibility in practice naturally depends on the sheer volume of submissions. Interestingly, and in contrast to the view that screening is inevitably a gate-keeping mechanism favoring established academics, some journals also entrust it wholly to junior researchers. One informant (editorial team #75) recounted how a journal editor recruited them as a screener early in their career:

And then it turned out that the…desk editor was really overwhelmed and bombarded with publications and at some point just reached out and said, “Look, I need someone to do screening for me. Like I basically just have too many submissions. Would you like to be a screener?” So not sort of an official editor, but working in that role of going, “Here’s a pile of papers, go through them. Tell them whether they are worth my while to even look at and then exclude the ones that don’t.”

One journal covered in our sample operated with a consistently collective approach to editorship at the time of our interviews, meaning that all editorial decisions including screening and final acceptance are made by a group rather than individuals, either via email or in virtual meetings. An informant (editorial team #64) emphasized the connection between the collective editorship model and the intellectual profile of the journal. The political content of the submissions, they argued, requires a distributed and particularly transparent process.

[T]he move in the field to want to matter to politics requires modes of publication that can handle the ethical charge of those works (…) there’s you know, been serious problems with the model of the lead editor, a single lead editor who then works with a managing editor who’s subordinate to them.

Such distribution of responsibilities of course is highly labor intensive and thus directly undermines the economizing function of screening. However, the increased workload is partly offset by the tightly knit structure of this particular epistemic subcommunity and by a consequently much smaller number of potentially unsuitable submissions.

### Assigning Reviewers

Many editors approach inviting reviewers as a challenge of intellectual match-making; that is, identifying “people who (…) [the author(s)] really should be in further conversation with, so that they can actually get a paper that contributes more specifically to STS” (editorial team #65). In principle, there is a common recognition among our informants that acting as a reviewer is a responsibility to the community and that peer review should ideally be a form of intellectual co-creation. Lack of institutional recognition for review work, however, has historically provided a powerful incentive against doing too much of it. We found that editors engage in diverse forms of work not only to cultivate a sufficient pool of available reviewers but to ensure that these reviewers perform their task under the right emotional, social, and epistemic conditions. If these are not actively created, review assignments—if they are accepted at all—risk degrading from constructive feedback to mere gate-keeping.

We have already mentioned one activity to achieve such favorable conditions, namely thorough screening of manuscripts, which is partly a gesture of respect toward reviewers. Aside from this, the challenges for editors in constituting a peer review gift economy significantly differ across journals; prestigious journals generally find it easier to recruit reviewers because of their visibility, while newer and less well-known journals cannot count on the journal’s prominence to attract referees. Statements by our informants, as well as recent research on publishing practices in other fields (Nästesjö 2021), suggest that this split is intensifying. For example, both employing institutions and senior colleagues actively encourage junior scholars to review only for journals they wish to publish in themselves (author and reviewer #14). Such advice reinforces an instrumental view of review labor as a means to selectively establish mutual obligation with a small range of particularly prestigious journals (rather than a community as a whole), thereby facilitating future submissions of one’s own.

Smaller and less well-known outlets often have to rely on alternative strategies to ensure the supply of review labor. One informant (editorial team #1) associated with a more recently founded journal explained how their involvement in the STS community over many years has resulted in the accumulation of a large number of favors owed to them personally by others. They try to convert these favors into reviewers. In other words, personal relations among a senior scholar and their colleagues are mobilized to create a reviewer pool from scratch.

But even editors of more prestigious journals spoke about the importance of reviewers being committed to the field/journal, which emphasizes that reviewers are not “fungible”—that is, some will write more useful and constructive reports than others. A number of editors also suggested that particularly established and well-known scholars are generally not as likely to accept review invitations as one would hope (editorial team #59, 64). This may of course be due to the fact that such individuals get a disproportionate number of requests to begin with. In response to such difficulties, many journals have expanded their editorial or advisory boards in recent years, in the hope that this will make the members more likely to take on handling of submissions and review requests.

The challenge of ensuring review labor is further complicated by the growing need for this labor to be suitably diverse in epistemic terms. To be sure, STS was already quite diverse in the 1980s, comprising such normatively opposed traditions as Mertonian sociology of science, SSK, Marxist critique of science, and actor-network theory. However, there were overall fewer active STS scholars and scholarly communication took place in a small range of journals. As a result of this coincidence between community and communication channels, scholarly debates were more generalist in orientation. Different conceptual traditions competed in providing mutually incompatible answers to a relatively limited set of questions: for example, is formal scientific discourse a faithful depiction of the underlying work, or is it a selective and self-serving representation? In such a context, the challenge for editors lay not so much in recruiting enough reviewers, but rather in adjudicating between them. To illustrate, *Social Studies of Science* used to aim for no fewer than five reviewers per submission up until the 1990s (author & reviewer #16). This commonly resulted in very contradictory reports and the consequent need for the editor to take a side—often at the risk of appearing biased in favor of certain approaches.

By contrast, given the growth and epistemic differentiation STS has undergone since then, fewer and fewer reviewers are able or willing to adopt a generalist outlook on submissions. The challenge of reviewer match-making increasingly is to achieve an epistemic fit between highly specialized submissions and reviewers who can offer informed opinions on them in the first place, as this informant notes:

And there may be just a handful of people that are really working on that specific thing from that specific angle, with those specific data gathering and analytical techniques, that when you go to find a reviewer, it’s either, well, “I don’t have the expertise for that,” or, (…) “I know who the author is, so I’ve already read the paper.” And (…) that suggests to me that the reading practices are getting narrower and narrower. (Editorial team #59)

Editors must of course walk a fine line between suitable epistemic fit and avoiding cronyism (Teplitskiy et al. 2018). Researchers are generally more predisposed to accept invitations to review manuscripts in areas they are working on themselves, since this turns the review activity into a form of labor that is immediately useful for the reviewers’ own research. Yet editors want to avoid having close colleagues reviewing one another’s work—a situation that becomes harder to detect as the field grows. A variation of this issue is that potential reviewers are known to the editor but by virtue of their specialization no longer feel committed to the particular journal or to STS as such (editorial team #67). In short, match-making between authors and reviewers requires an ever-greater knowledge of field dynamics on the part of editors, and, in fact, a significant editorial effort to create a sense of community. One informant (editorial team #59) fittingly spoke of their work as a constant ethnography of STS; that is, an effort to add a layer of meta-information that smooths the review process.

Interestingly, big commercial publishers try to facilitate reviewer match-making through various technological solutions. For example, they provide digital infrastructure that suggests suitable reviewers for manuscripts to editors and partially automates correspondence with them. An informant, however, described correspondence via commercial journal management software as a mechanistic, robot-like interaction, which makes researchers much more likely to reject the invitation to review (editorial team #59). While this particular journal is affiliated with a major publishing company, the editorial team intentionally abstains from using the automated communication functionality, instead aiming for “boutique interaction” with reviewers. Personalized correspondence is not just a matter of politeness and negotiating review deadlines, it is also crucial for getting feedback on reviewer competences. By contrast, commercially operated reviewer databases draw on keywords previously submitting authors have assigned to themselves to describe their expertise. Reviewers here are sourced from across the entire journal portfolio of the respective publisher according to a platform logic, thus making keywords lose any community-specific connotations. The actual epistemic fit becomes hard to assess even if keywords appear suitable for a submission, not to mention the difficulty of gauging the commitment of a potential reviewer to a given journal.

The notion of a good fit in assigning reviewers to manuscripts has in recent years been further complicated by considerations of representation. We have already emphasized that submissions especially to highly visible journals over the last 15 years have vastly increased. But while there are now overall more authors getting published, these are disproportionately from North American and European countries that historically dominate STS. Areas that do not yet have strong institutional capacities, or that have developed capacities in different languages and thus different publishing markets, are at risk of becoming even more marginalized in comparison. In reaction to this trend, a variety of journals with an intentionally geographical focus have been founded in the course of the last decades; for example, *Tapuya* and *East Asian Science, Technology and Society*. Some journals also have begun to adopt a dedicated policy for submissions from outside the Global North, where authors are paired with at least one reviewer from the same geographical region (editorial team #2, #6). Rather than simply a selection mechanism, the epistemic exchanges induced through peer review here serve as a vector to change regional research landscapes.

Finally, a relatively recent challenge for editors is managing submissions in relation to turnaround time. Before digital journal management systems became widely used, correspondence among editors and reviewers was based on email and letters. Editors generally tried to keep an eye on review times and sent reminders to reviewers but often without systematic tracking (author and reviewer #28; editorial team #10, #12). This created the possibility for manuscripts to “get lost” (author and reviewer #52). Journal management software nowadays keeps track of timelines and sends automated reminders to reviewers. As a general rule, many journals encourage reviewers to submit their reports within four weeks, although average review times are likely more in the area of six to eight weeks (this is not including the time required for editorial handling of submissions and review reports). Several commercial publishers, moreover, track and advertise average turnaround time on journal websites in the hope of encouraging more submissions. At the same time, our interviews—although not designed to investigate this issue systematically—do not suggest very significant differences in average review times across journals. This makes sense insofar as different journals face distinct but ultimately equally pronounced challenges. Very prestigious journals find it easier to recruit reviewers but also attract more submissions. Less well-established journals have fewer submissions to process but often have a harder time recruiting reviewers and less bargaining power in negotiating review deadlines.

### Interpreting Review Reports and Production

A completed set of reviews is often considered a second potential cut-off point after the initial screening, where editorial teams have to gauge how much work is still necessary to make a manuscript publishable and whether further investment of editorial resources and time is warranted. Such decision-making, however, is again imbued with efforts to organize and maintain community relations while containing centrifugal forces.

A first type of difficulty has to do with the intricacies of commensurating review reports (Lamont 2012; Espeland and Stevens 1998). For editors to make sense of reviewer recommendations, they not only need to substantively interpret the actual reports but also assess the status of the advice. Yet the fact that reviewers themselves are increasingly specialized and often unknown to the editors makes such a meta-interpretation difficult. Is a review actually based on a “proper” STS reading and understanding of the manuscript, and therefore trustworthy? And is a stellar review perhaps the result of too close a fit between author and reviewer?

[I]f I’m writing to somebody because of a topic I don’t necessarily have any sense whatsoever about how to read their reviews and what sorts of personal relationships there are. So I had a manuscript—a review came in a few weeks ago (…). It was a glowing, positive review. Sort of over-the-top positive. I have no idea whether the reviewer is best friends with the author. (Editorial team #67)

Another challenge arises when review reports are either polarized or particularly lukewarm. While historically declining numbers of reviewers per submission makes the former scenario somewhat less frequent than in the past, it still occurs rather commonly. In such cases, editors can make suggestions to the author for how to deal with the reports. For example, should they rather follow the contradictory comments of reviewer A or B? The exact level of editorial attention, however, is again constrained by their contingent workload (editorial team #49, #54).

A further consideration in editorial decisions is what use authors are likely to make of the review outcomes. In case of substantial revisions, it is not straightforward that authors are willing to fundamentally rework their submission. Especially where a confirmation of acceptance is required for a tenure review or a job application, they may simply decide to resubmit to another journal. Editors commonly take into account previous interactions with authors to assess the likelihood of such scenarios:

[Y]ou’ve got to make the call as to whether or not, within two rounds, the argument is basically going to get there or not. That’s probably one of the biggest zones where I end up exercising a lot of editorial judgement, (…) knowing the author, because I can see the author and I’m not blinded to the author, maybe having interacted with this author before, or maybe having, you know, seen the path of the paper so far, do I think that this paper is actually going to get revised in time, essentially? (Editorial team #69)

At all times, editors have to maintain cordiality in their communication with authors. This particularly goes for rejections, since authors may still be contacted with review requests in the future. One informant reported that they commonly edit overly harsh review reports before passing them on, but ideally in a way that is respectful of the reviewer’s opinion (editorial team #64).

Our material also suggests that attention to metrics and constraints related to funding models of scholarly publishing condition editorial decision-making practices at least to some extent. Several editors pointed out that upon applying for assignment of an impact factor for their journals, Clarivate Analytics initiated a review process but did not provide much detail about its exact workings. Indexation does appear to include desk research to assess whether a review process is in place as well as consideration of acceptance rates to gauge selectivity. However, especially the latter criterion can be difficult to meet for new journals that have not yet received many submissions in the first place. Looking back on a failed application for indexation, one informant (editorial team #70) reflected that it would be useful to adopt a stricter approach to interpreting review reports and so increase the percentage of “revise and resubmit” decisions, which formally count as rejections for Clarivate. For more established journals that already have an impact factor, metrics become an issue only when they noticeably drop (editorial team #62). All of the editors we interviewed emphasized that acceptance decisions are unaffected by metrics, but they also reported that publishers do provide them with unsolicited advice on how to increase impact factors. One such piece of advice is to systematically treat manuscripts submitted by particularly well-known authors as engines to increase citation rates.

Moreover, the OA funding model based on APCs (paid by individual authors) is a general concern for editors (editorial team #6, #17, #60, #38). Many worry that this will undermine their efforts at organizing and managing community relations in the sense that especially younger authors and authors from non-Western countries may find themselves without access to the necessary resources. Some journals try to counteract these dynamics by granting OA waivers to deserving authors. However, such corrective action is problematic, because it requires editors to mobilize additional funds and because it intertwines consideration of economic and intellectual aspects in editorial decisions.

An analytically relevant, final part of the editorial process is copy-editing. Use of a shared intellectual jargon is widely considered a crucial element of academic communities but is especially hard to master for younger and non-anglophone scholars. There are commercial editing services, but charges in the area of US$1,000 per article make them prohibitive for many scholars. Journals affiliated with large commercial publishers typically do have the possibility to outsource this labor to publisher staff. Since these typically have no familiarity with the jargon and conceptual traditions of the field, however, this can be ineffective. Editors therefore often take on this work themselves (editorial team #8, #59, #75).

## Conclusion

The tensions arising within the peer review economy of STS journals in recent years are reminiscent of accounts of gift economies that are gradually reconfigured through commodification layered on top of them (Gregory 1982; Tsing 2013). In the process, individual social actors are inscribed as participants in more than one political–economic framework, usually with the effect that the more formalized and often currency-based mechanisms of exchange destroy or at least undermine the original cycles of gift exchange. To be sure, academic communities were never simply given (Lievrouw 1989); they always exist only in an approximate form and require perpetual efforts at community building. However, publishing practices are changing in ways that require both increased editorial effort and partly new practices to organize and manage cycles of gift exchange. Our most significant insight is that curating a functional peer review economy requires a combination of two strategies that are actually in tension with one another.

On one hand, the growing competition for publications in a relatively small number of journals forces editors to economize review attention through various forms of triage, effectively making the ability to establish wholly new ties with submitting authors a finite resource. To the same extent, already established ties in the shape of personal acquaintance or previous interaction come to play a particularly important role; for example, in finding suitable reviewers but also in gauging whether authors can be expected to rework submissions to meet the expectations of journals. Our analysis suggests that leveraging personal ties in the review gift economy, while traditionally associated with bias and cronyism (Bornmann 2011; Teplitskiy et al. 2018), is to some extent an attempt to make optimal use of gift exchange in an increasingly fragmented intellectual formation whose members are incentivized to behave like individualistic economic agents.

At the same time, however, editors across journals are acutely aware of the need to expand traditional cycles of gift exchange and avoid overreliance on existing ties. This is not only a requirement to foster novel forms of knowledge but also to ensure the long-term sustainability of the peer review gift economy as well as its intellectual and geographical diversity. Paradoxically, then, editors need to temper the very forms of social and epistemic boundary drawing on which their curatorial strategies otherwise rely, albeit at the risk of wasting precious journal resources. Openness in editorial work after all means the possibility of disappointment; for example, when investment of attention and labor in a submission are not reciprocated or exploited, or when the very novelty of manuscripts requires a particularly significant investment before reciprocal action can be realized.

What alternative strategies for organizing and managing a scholarly gift economy can be imagined that are not based on differentiation and selection and thus on perpetuating the decollectivizing tendencies implied by academic career structures and publishing business models? We offer two starting points in what should ideally become a wider debate among STS scholars.

A first concrete opportunity is to fundamentally rethink notions of epistemic fit in reviewer match-making. Currently, it is common sense to aim for pairing specialized submissions with equally specialized reviewers. But while this encourages sophisticated exchanges along shared lines of inquiry, the resulting differentiation of the communal discourse undermines the establishment of further cycles of gift exchange. Editors could alternatively think about matching authors and reviewers with complementary or simply unrelated specializations to foster integration of new arguments in a more widely accessible discourse.

Second, we also propose community-led experimentation with new technologies for peer review, albeit in ways other than the simplistic technological fixes offered by commercial publication infrastructure. To be sure, infrastructural tools are useful to tackle well-defined issues like plagiarism (Horbach and Halfmann 2020), but they are of very limited use in assisting editors with more substantive tasks such as reviewer match-making. We specifically propose to think about technological possibilities for establishing new forms of gift exchange. A hint of what this might look like is the *Social Epistemology Review and Reply Forum*, where authors and reviewers for the eponymous journal are invited to have an open conversation after publication of an article based on their exchange during the review process. This not only connects writing of a review report to the possibility of authoring a digital publication but also establishes new relations of mutual indebtedness among scholars who are no longer separated by anonymity.
